# UNDERSTANDING THE ROLE OF A SOCIALLY ASSISTIVE ROBOT TO SUPPORT AGING IN PLACE

**DOI:** 10.1093/geroni/igac059.280

**Published:** 2022-12-20

**Authors:** George Mois, Lizandra Vergara, Afnaan Afsar Ali, Mimi Trinh, Wendy Rogers

**Affiliations:** University of Illinois Urbana-Champaign, Urbana Champaign, Illinois, United States; Federal University of Santa Catarina, Florianópolis, Santa Catarina, Brazil; University of Illinois Urbana Champaign, Champaign, Illinois, United States; University of Illinois Urbana-Champaign, Champaign, Illinois, United States; University of Illinois Urbana Champaign, Champaign, Illinois, United States

## Abstract

Communities may lack the infrastructure and required resources to support healthy aging and aging in place. Advancements in technology development such as socially assistive robots and artificial intelligence present new opportunities to support and meet the needs of older adults. For example, Misty is a socially assistive robot designed to allow customizability and adaptability to support and meet diverse user needs pertaining to tasks and activities. Our research aim is to understand if Misty can facilitate social interactions with older adults, control the home environment, and provide reminders. We are exploring older adults’ attitudes towards Misty through demonstrations of these activities. Through this research we provide insights pertaining to the facilitators and barriers in the acceptance and use of a socially assistive robot like Misty, to support healthy aging. Furthermore, our findings will provide guidance for design and implementation of artificial intelligence through socially assistive robots in a home environment.

